# The Name-Letter-Effect in Groups: Sharing Initials with Group Members Increases the Quality of Group Work

**DOI:** 10.1371/journal.pone.0079039

**Published:** 2013-11-13

**Authors:** Evan Polman, Monique M. H. Pollmann, T. Andrew Poehlman

**Affiliations:** 1 Wisconsin School of Business, University of Wisconsin-Madison, Madison, Wisconsin, United States of America; 2 Department of Communication and Information Sciences, Tilburg University, Tilburg, The Netherlands; 3 Cox School of Business, Southern Methodist University, Dallas, Texas, United States of America; Hungarian Academy of Sciences, Hungary

## Abstract

Although the name-letter-effect has been demonstrated reliably in choice contexts, recent research has called into question the existence of the name-letter-effect–the tendency among people to make choices that bear remarkable similarity with the letters in their own name. In this paper, we propose a connection between the name-letter-effect and interpersonal, group-level behavior that has not been previously captured in the literature. Specifically, we suggest that sharing initials with other group members promotes positive feelings toward those group members that in turn affect group outcomes. Using both field and laboratory studies, we found that sharing initials with group members cause groups to perform better by demonstrating greater performance, collective efficacy, adaptive conflict, and accuracy (on a hidden-profile task). Although many studies have investigated the effects of member similarity on various outcomes, our research demonstrates how minimal a degree of similarity among members is sufficient to influence quality of group outcomes.

## Introduction

We share our birthday with only one person in 365, yet in a group as small as 23 people, the chance for two people sharing a birthday is greater than 50%. Evidently, coincidental matches on otherwise personal and unique information are not as rare as they first sound. Perhaps even more surprising is how two strangers–after discovering they share a birthday–behave with one another. People are more likely to comply with a stranger’s request, feel more connected to them, rate them more favorably, and show them more cooperation–compared to strangers *ex novo* [1]–[4].

Such direct effects of sharing a birthday on friendly behavior among strangers are often explained by the work of Heider [5] who proposed that incidental similarities create a “unit relation” that promotes positive feelings and attraction toward others. Put differently, people have a tendency to prefer things that remind them of themselves. Notwithstanding the effects of sharing a birthday, similar results are produced among people who share initials. Termed the *name-letter-effect* [6]–[8], research shows that people’s choices are reliably influenced by the similarity between the letters of the choice and the chooser’s own name (for a review, see [9]). For example, people are disproportionately likely to work at companies, buy stocks, donate to charities, hold stronger attitudes to brands, and prefer consumer products with names that begin with the letters of their own initials compared to other letters [10]–[16]. As Nelson and Simmons [17] put it, “Toby is more likely to buy a Toyota, move to Toronto, and marry Tonya than is Jack, who is more likely to buy a Jaguar, move to Jacksonville, and marry Jackie” (p. 1106).

There have been other findings in this area as well, such as the effect of one’s initials on selecting a mate or place to live [18], [19], but a recent review has challenged these findings on the grounds that archival data used to support them is corrupted with cohort, geographic, and ethnic confounds, as well as reverse causality [20], [21]. It is thus informative to examine the name-letter-effect using methods that avoid those problems.

To be sure, the name-letter-effect has not just been observed in decisions, but in performance outcomes as well. People whose first names begin with C or D have lower GPAs and attend lesser quality graduate schools than people with other initials–presumably because people with initials that begin with C or D have less aversion to receiving those letters as grades in school [17]. Although recent studies have observed the role of the name-letter-effect in a range of meaningful behaviors, it remains to be seen whether the effect generalizes to a social and group context in which individuals–who share initials with other individuals–are charged with completing a group task. In essence, we focus on the question of whether shared initials among group members’ names tilt groups to perform better.

We were drawn to this issue because groups are omnipresent, and because such a study permits an opportunity to examine the name-letter-effect in a context that is not subject to recently documented confounds. Simonsohn [20], [21] demonstrated that findings based on archival data used to support the name-letter-effect are potentially spurious, but this does not preclude the existence of the name-letter-effect (i.e., absence of evidence is not evidence of absence; cf. *argumentum ad ignorantium*). Indeed, we consider the possibility that the name-letter-effect will manifest in an intra-group context, whereby group members who share initials with other group members have a predisposed advantage for securing joint outcomes. In this vein, the goals of this study include investigating the name-letter-effect in a social and group setting by using both field and laboratory methods (in contrast to archival methods) that are not subject to the recently documented confounds.

In this paper, we are proposing a type of “social sharedness” hypothesis [22], but it is a type of sharedness that has yet to be examined. Virtually all existing demonstrations of similarity among group members involve surface-level characteristics (e.g., age) or deep-level characteristics (e.g., attitudes). For example, groups with members who are similar with respect to age show more attachment to each other, whereas groups with members with similar attitudes communicate more with another [23], [24]. We sought, however, to examine whether the limits of similarity might extend beyond what the current literature suggests. Unlike surface- and deep-level characteristics, initials typically provide little if any information about others, and logically should play little if any role in group outcomes. Nonetheless, we propose that sharing initials with other group members gives rise to a “value-in-similarity” effect.

Research has shown that seemingly superficial and non-diagnostic similarities such as sharing birthdays, clothes, names, and even earlobes are sufficient to create meaningful, social bonds among people [4], [25]–[27]. As a result of these sudden “unit relations”, group members are more likely to feel connected to one another and show each other more cooperation [4]. What is more, “unit relations” provide a source of positive affect that can spread to other members via contagion [28] with the end result that motivation among members increases, and group outcomes improve [29].

In this respect, our hypothesis is consistent with a recent demonstration by Holland, Wennekers, Biljstra, Jongenelen, and van Knippenberg [30] of the impact of self-symbols on motivation. As Holland and colleagues noted, someone writing a paper might be more motivated if his or her initials are flashed on the computer screen. In support of this, a number of researchers have suggested that names are imbued with positive affect [31]–[35]. As such, people feel attachment to the letters in their names, and by extension, to those who share their letters. It is only a small step from there to posit that these now-activated positive thoughts and feelings about others spill over to influence group processes and outcomes (for examples, see [36]–[38]).

To our knowledge, there is only one extant study that shows a connection between the name-letter-effect and interpersonal behavior. In their study, LeBel and Campbell [39] asked participants in romantic relationships to rate the degree that each letter of the alphabet is pleasing, and how satisfied they feel in their relationship. Their results show that participants demonstrated a significant bias favoring their own and their *partner’s* initials. This finding is based on an earlier study that found that, much like how people favor the initials in their own name, people favor the initials in close others’ names [40]. Of import, LeBel and Campbell reported that the higher participants rate their romantic partners’ names, the higher they rate the relationship satisfaction with their partners (and, the lower likelihood that they breakup with their partners four months later). Thus, LeBel and Campbell observed the name-letter-effect in repeated interpersonal experiences with a close other–such that people’s relationship satisfaction with their partners is predicted by how much people like their partners’ initials.

In the present research, we extend the initial findings by LeBel and Campbell and provide a comprehensive examination of the role played by the name-letter-effect in an interpersonal context. Like LeBel and Campbell, we examined the influence of the name-letter-effect in outcomes that involve more than one person. However, unlike LeBel and Campbell, we measured outcomes at the group-level (e.g., group performance) in lieu of a single participant’s interpersonal judgment (e.g., relationship satisfaction). Although related, group outcomes may be considered a more complex measure of interpersonal behavior, insofar as unique features of groups (e.g., relational demography, intra-group biases, majority/minority influence; for a review, see [41]) contribute to interpersonal and collective outcomes above and beyond individual- and dyadic-level social psychological features [42]. But more than that, we examined what effects sharing initials among group members have for members in the same group who do not share initials. In other words, we examined whether group outcomes are the result of *just* group members who share initials, or of *all* group members, including the group members who are in groups with members who share initials, but who do not themselves share initials with other members. At a minimum, this should confirm the success of the name-letter-effect in influencing “unit relations.” However, the measurement of group outcomes enables something more. We will be able to test the positive contagion of resultant “unit relations” that potentially underlies the relationship between the name-letter-effect and group outcomes.

Thus, although scholars have investigated whether people’s initials influence their choices [20], [43], we ask a different question: Do group members’ initials influence their joint outcomes (i.e., intra-group behaviors) with others who share their initials? In this regard, we carried out two studies, one in the field (Study 1) and another in the laboratory (Study 2). The field study was conducted in the context of self-managed student project groups, and sought to examine the relationship between groups’ proportion of members who share first name initials and group outcomes, such as performance, collective efficacy, and adaptive conflict. The laboratory study sought to extend these findings by manipulating the number of group members who share first name initials and then measuring group accuracy on a hidden profile task. In all, we expect groups with members who share initials to surpass groups with members who do not share initials. Together, these studies suggest a psychological connection between the name-letter-effect and interpersonal, group-level behavior that has not been previously captured in the literature.

### Ethics Statement

For the pair of studies presented, we obtained behavioral research ethics board approval from Cornell University (Office of Research Integrity and Assurance) and New York University (University Committee on Activities Involving Human Subjects). Participants gave written informed consent prior to participation, and received a written debriefing at the end of the study session. No minors or children were involved in our studies.

## Study 1

### Participants and Procedure

This study was conducted in an undergraduate course in which students complete a major group project (worth 40% of students’ final grade) over the duration of a 15-week semester. In particular, 262 undergraduate students were randomly assigned to 72 project groups consisting of three to five members. Each group was charged with examining a topic within organizational behavior (e.g., job satisfaction, employee motivation, leadership) and then examining that topic within the context of an actual organization. Two weeks before the group project was due (and after sufficient time for group members to learn each other’s names; specifically, after 7 weeks), students responded to items measuring collective efficacy and adaptive conflict, in addition to demographic information. Each of these measures is described below.

### Measures

#### Collective efficacy

Following recommendations made by Bandura [44], we measured levels of collective efficacy by providing each member of a group with nine performance benchmarks, specifically, to earn 100%, 98%, 96%, 94%, 92%, 90%, 85%, 80%, 75% (e.g., “How confident are you that your group will earn a 94% on the final project?”). The ratings were made on a continuous 100-point scale (0 = *not at all certain*; 100 = *absolutely certain*). Consistent with the procedures used in previous research (e.g., [45]), the level of collective efficacy was operationalized as the sum of the rating scores across the nine performance levels (*M* = 784.61, *SD* = 125.54), and showed strong within-group agreement (*r*_wg_ = .83; ICC1 = .32).

#### Adaptive conflict

Adaptive conflict focuses on strategic and logistical issues such as scheduling deadlines and the division of labor [46], [47]. Referred to sometimes as *process conflict*, it is distinguished in theory from *relationship conflict* which refers to interpersonal incompatibilities among group members, including personality differences, and *task conflict* which refers to disagreements among group members about the content of the task being performed [48]. Specifically, students responded to three items (e.g., “How much conflict is there in your group about task responsibilities?”) from 1 (*none*) to 5 (*a lot*) on a validated scale (α = .93; *M* = 1.56, *SD* = 0.52) created by Jehn and Mannix [49] that showed strong within-group agreement (*r*_wg_ = .70; ICC1 = .86) and has been used in other research to measure adaptive (i.e., beneficial) conflict [50].

#### Group performance

Each group of students was required to produce a final written report, detailing their findings. The course instructor graded the papers on a scale of 0–100 points (*M* = 92.58, *SD* = 3.57). Each group handed in one paper, and all members received the same grade. The course instructor did not know the purpose or hypotheses of our study.

#### Demographics

As a control variable, we also measured surface level diversity–the distribution of within-group ethnicities. Because Simonsohn [20] claims that some of the name-letter-effect findings are the result of ethnic confounds (e.g., people in ethnic groups are more likely to marry within their own groups, and ethnic groups have different distributions of names and initials), we include this variable to control for the possibility of ethnic-matching behavior. Specifically, we measured surface level diversity by adding the squared proportions of each ethno-racial category that comprise a group, and subtracting that number from one (cf. [51]).

## Results and Discussion

**Figure 1:**
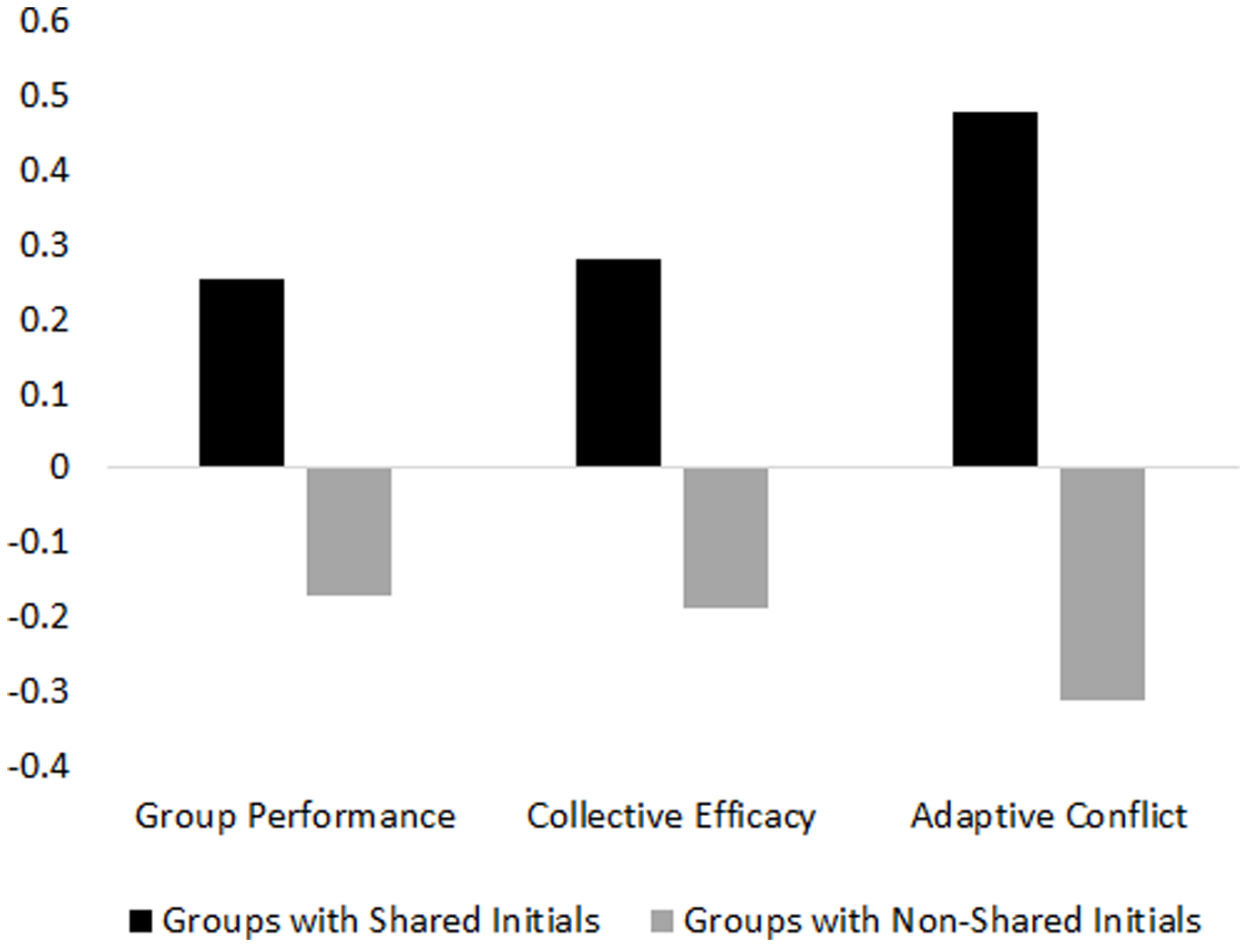
Group outcomes according to groups with members who share initials and groups with members who do not share initials (results have been z-transformed).

We regressed each of our dependent measures on the proportion of group members who share first name initials. In order to account for groups that might have more than one pair of members who share initials (e.g., a 5-person group might include: Emma, Elizabeth, Michael, Michelle, and Tara), we added the squared proportion of each unique initial found in a group. This index is perfectly correlated with the raw proportion of members who share initials. However, using this index allows us to include and calculate a proportion for groups that have more than one pair of members who share initials–which would otherwise be excluded because a raw proportion cannot be calculated for such groups. In our sample, the proportions ranged from 0 to.625; the average proportion was.15. As expected, groups with a higher proportion of members who share initials exceeded groups with a lower proportion of members who share initials on every dependent measure, and controlling for surface level diversity and number of members in a group did not alter the results (see Table 1 and Figure 1 for a detailed description of the results). Although there is no normative reason for why members’ sharing initials should have any impact on group outcomes, these outcomes were nonetheless associated with the sharing of initials among group members. Thus, this study provides initial evidence that sharing initials among group members is related to the quality of group outcomes.

**Table 1:** Results of regressions predicting the effects of sharing intials on group performance, collective efficacy, and adaptive conflict (after controlling for surface level diversity and number of group members).

	Standardized Coefficient (Beta)		
	Group Performance	Collective Efficacy	Adaptive Conflict
Proportion of members who share initials	.27*	.26*	.32*
Surface level diversity	−06	−09	.07
Number of group members	−12	.04	.07
R^2^	.06	.08	.14

* *p*<.05;

** *p*<.01;

*** *p*<.001.

It is worth noting what effects sharing initials among group members have for members in the same group who do not share initials. Put differently, it is an open question as to whether an increase in positive group outcomes is squarely the result of group members who share initials, or of all group members writ large. Our data suggest that “unit relations” are contagious and spread to all members. Among groups with members who share initials, we observed no significant difference in collective efficacy or adaptive conflict between members who share initials (*M*_collective efficacy_ = 775.11, *SD*_collective efficacy_ = 195.57; *M*_adaptive conflict_ = 2.01, *SD*_adaptive conflict_ = 0.94) and members who do not share initials (*M*_collective efficacy_ = 790.79, *SD*_collective efficacy_ = 253.44; *M*_adaptive conflict_ = 1.74, *SD*_adaptive conflict_ = 0.81), *t*s<1.5. Thus, we observe that in groups with similar members, assessments such as collective efficacy and adaptive conflict are the same between similar and dissimilar members, suggesting that positive group outcomes are the result of all members (not just the similar members) profiting from “unit relations.” That is, similarities among some members in a group are sufficient to improve group outcomes–in that in groups with similar members, the dissimilar members behave at the same high levels as the similar members. These results are encouraging because they suggest that the positive contagion of “unit relations” helps explain the relationship between the name-letter-effect and group outcomes.

A limitation, however, of Study 1 is that groups were not formed with the intention to match initials, so the correlational nature of this design precludes causal inferences. In this regard, we carried out a second study to test whether groups designed to include members who share initials have an advantage over groups designed to not include members who share initials–choosing as our measure for group performance the most concrete instrument we could find. Specifically, we expect groups with members who share initials to perform better on a hidden profile task–a widely used measure among small group researchers to examine the degree that groups pool information and identify a correct solution to a problem [52], [53]. The results of this study could shed more light on whether groups with members who share initials out-perform groups with members who do not share initials.

## Study 2

### Participants and Procedure

Three hundred and ten undergraduate students participated in a class exercise on groups and teams. In a departure from the previous study, we created 54 groups, consisting of four to six members, with the pre-planned intention that half of the groups comprise two members (and only two members) who share first name initials (*n* = 27), whereas the other half of the groups comprise members who do not share first name initials (*n* = 27). In the former condition, the proportion of members who share initials ranged from.50 to 1.00; the average proportion was.69.

Before beginning the exercise, participants were asked to introduce themselves to each of their group members, and write their names on a form that we provided. Next, participants completed the murder mystery decision task from Stasser and Stewart [54]. Specifically, participants read a series of interviews from a fictional homicide investigation. Of import, contained in the interviews are clues that are critical to solving the mystery. In particular, the clues incriminate three suspects, Eddie, Billy, and Mickey; yet exonerate two of the suspects, Billy and Mickey. Although Eddie is the obvious culprit, correctly identifying Eddie is relatively difficult for a group when the clues hinting to Eddie’s culpability and to Billy’s and Mickey’s innocence are randomly distributed among members in such a way that members do not have the same clues as other members. That is to say, in each group, members received *unique* clues that incriminate Eddie and exonerate Billy and Mickey, yet the *same* clues that incriminate Billy and Mickey. Thus, collectively, group members had all of the necessary information to solve the crime but the solution to the mystery was not likely to be discovered unless the unique, non-redundant information was discussed. As research has shown, this is not often the case–rather, group members have a tendency to focus on information that all members have in common (e.g., clues that incriminate Billy and Mickey) in contrast to exchanging unique information (e.g., clues that incriminate Eddie; [55]). However, if all the evidence is considered and shared, then it should be clear that Eddie is the guilty suspect and has both the motive and the opportunity to commit the crime.

After reading the materials, groups were given 20 minutes to discuss the murder case and make a group decision. Each group was asked to decide on the suspect that it believed most likely committed the murder. The decisions that groups indicated comprised our dependent measure, group accuracy.

## Results and Discussion

We predicted that groups with members who share first name initials will be more likely to reach the correct solution than will groups with members who do not share first name initials. Consistent with our prediction, 70% of groups with members who share initials correctly identified Eddie as the suspect, a value that is reliably above chance, *z* = 2.93, *p*<.01, and more importantly, significantly greater than the 41% of groups that identified Eddie and have no members who share initials, χ^2^(1, *N* = 54) = 4.80, *p*<.05, η^2^ = .08. Thus, the ostensibly superficial manipulation of creating groups based on members’ names seemed to have a considerable impact on the actual behavior of groups. Those groups with members who share initials were 70% more likely to identify the correct answer than groups with members who do not share initials. The results confirm the potent influence that sharing initials among members can have on group outcomes.

## General Discussion

The present pair of studies was designed to examine whether incidental similarities among group members influence group outcomes. Specifically, we were interested in situations in which group members share initials with other group members. Such similarities provide no relevant information about group members, and should not, in the abstract, play a role in increasing the quality of group outcomes. Nonetheless, we found that grouping members according to their initials can significantly increase a breadth of group outcomes as varied as performance, collective efficacy, adaptive conflict, and accuracy.

This research makes three primary theoretical contributions. First, our findings extend our understanding of the influence of the name-letter-effect. Prior work has demonstrated that people’s choices, attitudes, and preferences uncoincidentally resemble (i.e., share) the letters in their own names [43]. Our work demonstrates that people’s group outcomes are also sensitive to the name-letter-effect, such that sharing initials with others has broad consequences. We find that groups with members who share initials out-perform groups with members who do not share initials. More to the point, we extend the current findings of the name-letter-effect to a social, interpersonal context. Understood in this way, group outcomes involve not just the self but the consideration of others–and we find that others in a group who share initials with others predispose the group to waxed levels of collective efficacy, adaptive conflict, performance, and group accuracy, a type of interpersonal name-letter-effect unto itself.

Second, this work deepens our understanding of groups. Rather than living in isolation, people are members of different social groups (e.g., family, company, religion). And a large body of literature studies intergroup relations, in particular out-groups–the groups to which people do not belong and foster contempt, opposition, and competition among people [56]. We contribute to this literature by highlighting an easy way to facilitate social coordination and foster social bonds among in-group members, but also between in- and out-group members. By sharing a first name or initial with an out-group member, people may better coordinate their behavior and bond with out-group others–not unlike the effects of taking out-group others’ perspective and subsequently feeling more similar to them [57].

Third, this work contributes to extant research highlighting the importance of implicit, nonconscious influences in interpersonal and group settings (e.g., [58], [59]). Researchers interested in psychological processes underlying negotiation have addressed the potential role of priming and other nonconscious, automatic processes [60]–[62] but no prior research has studied how the letters in one’s name might incidentally influence interpersonally relevant decision making, such as the names of one’s negotiation partner or client. Future work should study these relationships and even consider the broad implication that sharing initials with others may improve negotiation outcomes vis-à-vis more integrative agreements and better client relations.

In light of the recent research that has challenged the notion that people’s decisions such as where to live, whom to mate, and what career to choose are influenced by the letters in people’s names [20], [21], an important question remains: why might group outcomes be more sensitive to the name-letter-effect compared to individual decisions?.

One possibility is the ease with which “unit relations” are created among people. In contrast, individual decisions are less susceptible to the benefits of “unit relations.” In fact, “unit relations” arise *prima facie*, in the sense that they are instant bonds among strangers who, aside from sharing something incidentally similar such as a name, have little other information about each other. In major life decisions, however, people have access to lots of information. For example, Louis might be more likely to choose to live in St. Louis relative to other cities, but, if Louis knows that in 2013, the city of St. Louis ranked number 12 on the Forbes list of top 20 most miserable cities to live in America [63], he might be equally less likely to choose to live there compared to, say, Jack. In other words, name-letter-effects, although statistically robust, are quite small in comparison to other determinants of decision making. When people have lots of information, it is not likely that letters in names will have a large influence on their choices, but, when people have less information about their environments, then we might expect letters in names to exert relatively more influence–such as the case among newly formed groups that are comprised of members who do not know each other very well.

In this vein, our research revealed that it was fruitful to combine research on the name-letter-effect with research on groups. In the context of groups, many studies have investigated the positive effects of member similarity on group performance, collective efficacy, conflict, and information sharing–among other outcomes such as trust and morale [48], [64]–[68]. In addition, research has investigated the positive effects of member diversity (in contrast to similarity) on group outcomes–the so-called “value-in-diversity” hypothesis (e.g., [69], [70]). For example, groups with diverse members show more creativity and innovation than groups with similar members [71], [72]. Despite that similarity and diversity are sometimes a boon or a bust among groups, the findings are not always equivocal–as Ayub and Jehn [73] put it in their recent review of group diversity, “the effects of diversity are noticeably associated with other factors that make it good or bad” (p. 13). Thus we suspect that although sharing initials with group members leads to some positive outcomes, it may also lead to some negative outcomes in which member similarity has been shown to interfere with group performance (e.g., creativity). In all, the present research contributes to this work by demonstrating how minimal a degree of similarity among members is sufficient to influence the quality of group outcomes. Despite the research is ripe for investigating the effects of incidental similarities in social and intragroup contexts, there are issues to bear in mind.

Although our findings establish an important link between the name-letter-effect and group outcomes, future work remains with respect to understanding the underlying mechanisms that explain why groups with members who share initials out-perform groups with members who do not share initials. Presumably, groups absorb the positive affect that results of “unit relations,” yet this claim remains unclear. Still, the fact that group outcomes can increase from such a small and easy-to-use manipulation is intriguing and interesting in its own right.

In closing, we do not want to overstate our findings. The conclusion that groups with a higher proportion of members who share initials fare better than groups with a lower proportion of members who share initials seems remarkable enough to beg additional verification. As suggested by Pelham and Carvallo [43], there are a number of likely moderators of the name-letter-effect, variables that may increase or decrease the effect of sharing initials among group members on group outcomes. In this vein, while conducting the current research, we collected two samples of groups where we did *not* observe a name-letter-effect on group outcomes. In these samples, groups with members who shared initials performed to the same extent as groups with members who did not share initials. It is not clear, then, when the name-letter-effect will influence group outcomes and when it will not. Notably, in these samples, we did not have measures of surface level diversity; and one sample comprised of professional athletes, whose level of expertise may be expected to crowd out the name-letter-effect. Because of this variance, we are open to the possibility that the net effect of sharing initials with other people is in favor of few findings, than in significant findings. This is perhaps the most important finding of our research. Still, we believe it is worth experimenting with incidental similarity cues, such as names. Organizations or individuals who form groups can tailor the members such that the likelihood of incidental similarities among members is maximized. Evidently, matching initials of potential colleagues such that Tajfel is paired with Turner, or Schachter is coupled with Singer, is an easy way to potentially increase the quality of groups.
